# Effect of New 2-Thioxoimidazolidin-4-one Compounds against *Staphylococcus aureus* Clinical Strains and Immunological Markers' Combinations

**DOI:** 10.1155/2022/6720241

**Published:** 2022-07-13

**Authors:** Hanan Tariq Subhi, Hiwa Ramadhan Fatah, Hanaa Ali Muhammad

**Affiliations:** Department of Biology, Faculty of Science and Health, Koya University, Koya KOY45, Kurdistan Region–F.R., Iraq

## Abstract

Although the structure-activity relationship indicates that the 4-thioxoimidazolidin ring is essential for antibacterial activities and pharmaceutical applications, there were no enough studies on the derivatives of this compound. Evaluating the new hydantoin compounds C5 (3-((2-bromobenzylidene) amino)-2- thioxoimidazolidin-4-one) and C6 (3-((4- methoxybenzylidene) amino)-2-thioxoimidazolidin-4-one) that were prepared against clinical *Staphylococcus aureus* isolates for antibacterial, antibiofilm, and antihemagglutination activities is the aim of this study. Therefore, the potential clinical resistance of the strains was evaluated by their ability to form biofilms, antibiotic resistance, and agglutinate erythrocytes macroscopically and microscopically; besides, the bacterial biofilm was screened for any association with the patient's serum immunoglobulin levels and complements. Despite the effective concentration for C5 and C6 compounds, which is ≤ 31.25 *μ*g/ml, the reduction rate is not concentration-dependent; it depends on the molecular docking of the hydantoin compounds. Hence, the effect of the minimal inhibitory concentrations (MICs) is variable. In this study, the results for the compounds (with the concentration of 31.25–62.5 *μ*g/mL for C5 and 62.5–125 *μ*g/mL for C6) significantly manifest the antibacteria, antibiofilm, and antihemagglutination effects against the virulent strains of *S. aureus* due to the high percentage of biofilm inhibition that was caused by the new hydantoin compounds. Besides, time-kill kinetics studies showed that these compounds pose bactericidal action. Overall, this study revealed that the new hydantoin derivatives have an interesting potential as new antibacterial drugs through the inhibition of bacterial adhesion. The infections of these isolates activate the complement system through the lectin pathway. Nevertheless, these compounds can be improved in order to be used at even lower concentrations.

## 1. Introduction

Infectious disease persistence is the leading cause of death, particularly in developing countries; hence, the number of multi-drug resistant strains of microbial pathogens has increased [1]. *Staphylococcus aureus* is one of the crucial pathogenic bacteria that needs to be studied in healthcare or in the community as it is a major cause of both nosocomial and community-acquired infections since it overcomes the immunity and invades deeper tissues; consequently, the infections no longer respond to antibiotics treatment [2, 3]. The biofilm of Staphylococci is the most complicated type of infection as it possesses antimicrobial resistance due to the metabolic and physiological activities of bacteria which are different in the layers of the biofilm [4]. Besides, biofilm is the most common Staphylococci virulence factor, which is responsible for many chronic diseases and immune system evasions [2, 5, 6]. In effect, bacteria organized in biofilms are far more difficult to eliminate than planktonic bacterial infections, as some *Staphylococcus aureus* produce lineages that contribute to strong biofilm formation [7]. The reason for the dominance of diseases caused by this bacterium is the mode of transmission from the nasal mucosa membranes and skin to the broken tissue. Furthermore, *S. aureus* has the ability to establish biofilms on medical instruments such as catheters and other devices, which are difficult to be removed [8]. Thus, *S.aureus* is well known as being notorious [9]. Microorganisms have some advanced approaches to resist the toxic side effects of antibiotics and other drugs. Currently, pathogenic bacteria do not only possess the resistance mechanism of beta-lactamase enzymes, but these isolates often produce multiple enzymes that cause serious therapeutic problems in many parts of the world [10]. The most recent studies focus on investigating new drug compounds to treat a variety of illnesses; for that to be achieved, new synthetic pathways or structural modifications of the existing molecules and therapeutic drugs need to be studied in order to improve more effective drugs and to yield fewer toxic traits, which are frequently synthetic via modification or molecular alteration using bioisosteres [11]. Hydantoin is an organic compound with the same heterocyclic ring (imidazolidine); the creation of imidazolidinone has been studied intensively for its important pharmacological characteristics, fungicidal, anti-HIV, antitumor, hypolipidemic, anti-inflammatory, antihypertensive, antiarrhythmic, antimicrobial, and as effective disinfectant characteristics with broad antimicrobial activities, which are affected by the gradual release of oxidizing halogen in water [12]. Imidazoles1,3-C_3_N_2_ ring possesses multiple substituent features and biological molecules, such as histidine and histamine, in a purine structure [13, 14]. Hypnotics drug was the first hydantoin that has been used to treat chorea and epilepsy [15]. Then it was improved to be used more in medicine, for instance, by changing the N-3 hydrogen atom to the methyl group to reduce toxicity [16, 17]. Recently, hydantoin derivatives have been used as therapeutics and nontherapeutic products; for example, N– nervous system, NO_3_–antiepileptics, NO_3_A–antiepileptics, and NO_3_AB–hydantoin derivatives [18, 19]. Discovering chemotherapeutic agents has flicked the essential part to prevent and control such diseases. These agents are secluded either from the cover of organisms that are identified as antibiotics or chemically prepared via chemists [20, 21]. Thus, new hydantoin compounds can be used properly as antimicrobial agents, especially in emergency cases when pathogenic bacterial strains are resistant to all known antibiotics. The most promising compounds that have been identified by screening were tested against multidrug-resistant *Staphylococcus aureus* clinical isolates. In this study, the new hydantoin was applied as an antibiofilm drug and then the effect of these new compounds on human erythrocytes' agglutination and the interaction between the antibodies and the complement system were revealed; in the current study, *S*. *aureus* is included as a common example.

## 2. Materials and Methods

### 2.1. Synthesis and Characterization of the New Hydantoin Derivatives

Precursors were prepared according to [22–25]. The new hydantoin derivatives were prepared by synthesizing2-arylidene hydrazine carbothioamide compounds (S5, S6), and then 3-arylidene-amine-2- thioxoimidazolidin-4-one (C5 andC6).  Table 1 illustrates the physical properties of S5, S6, C5, and C6.

Calculating the uncorrected melting point and Fourier Transform Infrared Spectroscopy (FTIR) were used for the characterization, and Shimadzu (Japan) FT-IR was used for the analysis. The structure of these compounds was determined by proton nuclear magnetic resonance (1H-NMR) spectroscopy.

### 2.2. Biological Evaluation

#### 2.2.1. Collection of Samples

The clinical samples of nosocomial infections were collected from 3 hospitals in the Baghdad city of Iraq. Among the 60 isolates, 12 of them were *S. aureus.* The identification was performed based on conventional biochemical tests, Gram stain, morphological characteristics of the colonies on mannitol salt agar and blood agar, Api Staph strip reading, and confirmed by VITEK 2 compact system (BioMerieux, France) [26, 27].

#### 2.2.2. Biofilm Formation

The presence and the activity of the biofilms were measured by a quantitative method. The clinical isolates of *S. aureus* were screened in the current study for their capability to form biofilms via Micro titration Plates Approach (M.T.P) as stated by 28. *S. aureus* culture was grown overnight in a brain heart infusion broth with 1% (w/v) glucose at 37°C which was diluted to 1 : 20 in the brain heart infusion broth (BHI) containing 1% glucose; then, 200 *μ*l broth culture was added to each 96 well-flat bottom polystyrene titer plate and the inoculated well plastic plate was incubated at 37°C for 24 hours. The next day, the broth culture was discarded and the wells were gently washed twice with 200 *μ*l of distilled water and then dried. This was followed by adding 200 *μ*l of 0.1% safranin to each well for biofilm staining. The plate was kept for 5 minutes at room temperature. Each well was washed twice with 200 *μ*l of distilled water and then left to dry at room temperature. Finally, the biofilm was quantified by bacterial biomass adherence to the floors of the plastic plate using the microplate ELISA reader at 490 nm optical density (OD) for each well 25 (Table 2). The wells to which the sterile nutrient broth lacked bacterial cells was included and served as a control; the OD values for these wells were subtracted from the experimental readings.

Biofilm formation ability = OD of the tested sample—OD of quality control.

### 2.3. The Sensitivity of Isolates to Ten Antibiotics

According to the Clinical and Laboratory Standards Institute (CLSI) standards, sensitivity to Ciprofloxacin (CIP 10 *µ*g), Levofloxacin (Lev5 *µ*g), Amikacin (AK 30 *µ*g), Topromycin (TOB5 *µ*g), Piperacillin (PRL 30 *µ*g), Amoxicillin/Clavulanic acid (AMC 20/10 *µ*g), Imipenem (IPM 30 *µ*g), Azithromycin (AZM30 *µ*g), Ceftazidime (CAZ30 *µ*g), and Vancomycin (VA 30 *µ*g) disks was measured. Bio analysis (Turkey) were carried out on Mueller-Hinton agar plates using the diffusion method [29].

### 2.4. Immunoglobulin and Complement Determination

Single radial immunodiffusion (SRID) of [30] was used to determine the serum concentration (immunoglobulin and complement components). According to the producer, 5 *μ*l serum from the patient was used for each test. Endplates were used to determine IgG, IgM, IgA, C3, and C4. The plates were partly opened for 5 minutes to eliminate moisture droplets, then closed tightly, and left on the bench at room temperature on a level surface for 48–72 hours. The immunoprecipitation ring diameter was measured to the nearest 0.1 mm by a specific ruler.

### 2.5. Hemagglutination Assay

Blood samples were collected from patients who suffer from *S. aureus* infections. The blood with heparin was used to retrieve erythrocytes; 5 ml of blood was added to 45 ml of saline solution and then centrifuged twice at 2500 rpm for 10 min. Next, 1% erythrocyte solution was prepared; 100 *µ*l of the pellet was resuspended in 10 ml saline solution. Hemagglutination assay was performed using the previously described method [23] with some modifications. Briefly, the *S. aureus* culture grew in fresh tryptic soy broth (TSB) supplemented with 0.25% glucose at 37°C for 18 h; the bacterial cells were harvested by centrifuging and washed with phosphate-buffered saline (PBS) containing 0.1% bovine serum albumin (BSA). The cells were resuspended in saline, adjusted to 3.0 McFarland standard in PBS with 0.1% BSA, which correlated with∼9 × 10^8^ bacteria/ml. Each cell suspension (100 *µ*l) was added to 96 well (U-shaped) microtiter plates, and then 100 *µ*l of the 1% erythrocyte solution (in PBS with 0.1% BSA) was added to each well; to ensure the thorough mixing of the bacteria and erythrocytes, the total volume of each well was pipetted in and out with a micropipette. The wells were incubated at room temperature for 2 h. The hemagglutination titer was evaluated macroscopically; erythrocytes that appeared to be negative for hemagglutination were evaluated microscopically. All experiments were done with 3 duplicates. The positive result appeared as a uniform thin film of erythrocytes covering the bottom of the well, while the negative results appeared as a compacted red button of sediment erythrocytes.

### 2.6. *In Vitro* Biocompatibility Evaluation

#### 2.6.1. Antibacterial Efficiency

Minimal Inhibitory Concentration (MIC) values were determined using the standard broth dilution method, i.e., serial two-fold dilutions (range from 15.62 mg/ml to 500 mg/ml) of the new compounds [((4- hydroxyphenyl ethylidene) amino)-2-thioxoimidazolidin-4-one] that were prepared in the BHI broth [29]. *S. aureus* were grown in the BHI broth medium (positive control); the negative control contained only the BHI broth without the bacteria, followed by incubation at 37°C for 24 hr in aerobic conditions. The MIC values were defined as the lowest concentration of the new hydantoin compound that inhibited 100% of *S. aureus* growth compared with the negative control.

#### 2.6.2. Time-Kill Test

The nutrient broth medium with *S. aureus* cells at a density 1.5 × 108 CFU/ml and new hydantoin compounds C5 and C6 at concentrations of 1×, 4×, 8, and 16× the minimum inhibitory concentration (MIC) was incubated at 37°C. Control was only the broth inoculated with the same cell number of bacteria. Each treatment as well as the control (100 *μ*l) was inoculated onto nutrient agar plates, and measuring colony counts were at 0, 6, 12, and 24 h. The kill measurement and half-maximal inhibitory concentration (IC50) were calculated by plotting the viable colony counts as log10 (CFU/ml) versus time [31].

#### 2.6.3. Antibiofilm Assay

To evaluate the efficacy of the new hydantoin derivative compounds in interrupting *S. aureus* biofilm formation, the M.T.P assay was carried out accordingly by [32, 33] using 96 well-flat bottom polystyrene titer plates. Individual wells were filled with 180 *µ*L BHI broth containing 1% (w/v) glucose followed by inoculation with 20 *µ*L of overnight bacterial culture. To this, 100 *µ*L hydantoin compounds were added in concentrations of 31.25, 62.5, 125, 250, and 500 *µ*g/mL and covered by parafilm along with control (without hydantoin compounds) and incubated at 37°C for 24 h.Then, the wells' content was removed, washed twice with sterile water, and left to dry at room temperature for 15 min. To evaluate the adherence of sessile bacteria, they were stained with crystal violet (0.1%, w/v) and left for 20 min. The excessive stain was removed by deionized water and kept for drying. Furthermore, dried plates were washed with 95% ethanol and then optical density was determined using a microtiter plate reader (BIORAD) at 595 nm. The biofilm inhibition rate (Percentage Inhibition %) was calculated using the formula:

% Biofilm Inhibition = 1– (Sample optical density/Control optical density)×100 [34].

### 2.7. Effect of the New Hydantoin Compounds on Hemagglutination

The activity of the two new hydantoin derivatives on hemagglutination was assessed correspondingly as previously described above [29]. The new hydantoin derivatives were added to each well at subMICs concentrations. The experiment was performed in triplicate. Finally, the result was compared with the control samples, i.e., wells consisting of hydantoins alone and wells without hydantoins.

### 2.8. Statistical Analysis

Statistical Analysis System - SAS (2012) program was used to analyse the difference between the study factors [35].

## 3. Results and Discussion

### 3.1. Characterization of the New Compounds (C5 and C6)

**Figure 1:**
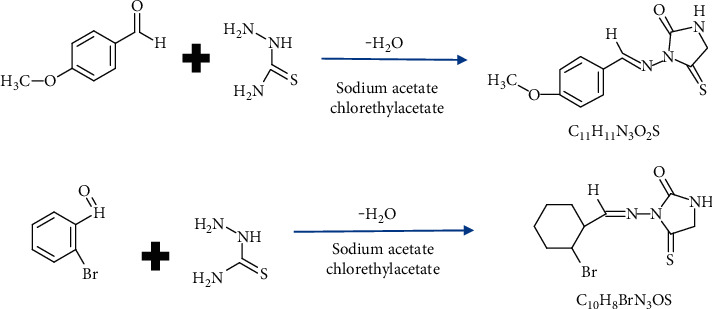
Synthetic diagram of new compounds (C5 and C6) [25].

Those compounds that were synthesized are described in Figure 1.

### 3.2. Fourier Transform Infrared Spectra of the New Hydantoin Derivatives

The formation of the new compounds was confirmed by FTIR.

### 3.3. FTIR Spectrum of S5 and S6

**Figure 2:**
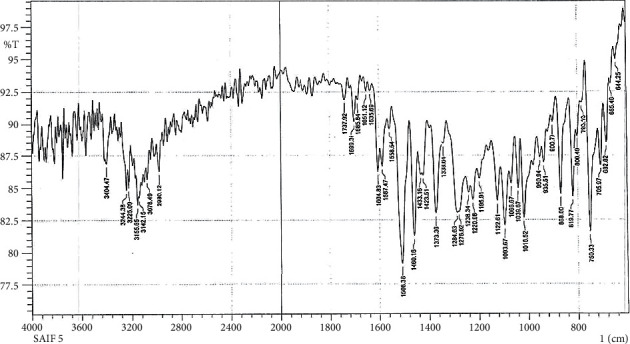
Fourier Transform Infrared Spectrum of S5.

**Figure 3:**
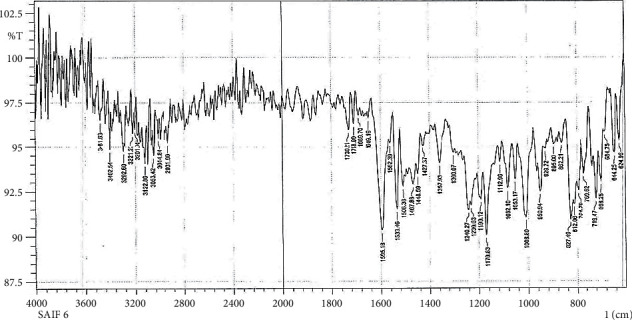
Fourier Transform Infrared Spectrum of S6.

In Figures 2 and 3, the FTIR spectra of S5 and S6 exhibit peaks at 3404 cm^−1^, 3244 cm^−1^, 3402 cm^−1^, and 3292 cm^−1^, respectively, denoting *υ*as and *υ*s (NH2) vibrations [30, 32, 36]; the absorption band of *υ* (*C*=*S*) seems to peak at 1093 cm^−1^ and 1082 cm^−1^, respectively [37, 38].

### 3.4. FTIR Spectrum of C5 and C6

**Figure 4:**
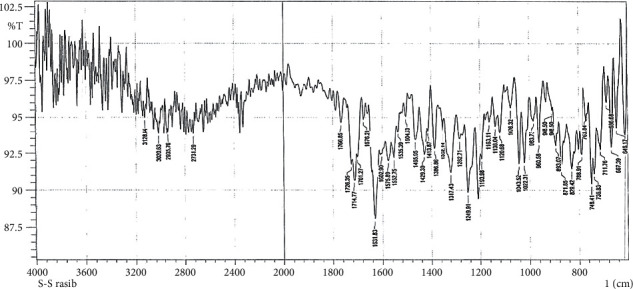
Fourier Transform Infrared Spectrum of C5.

**Figure 5:**
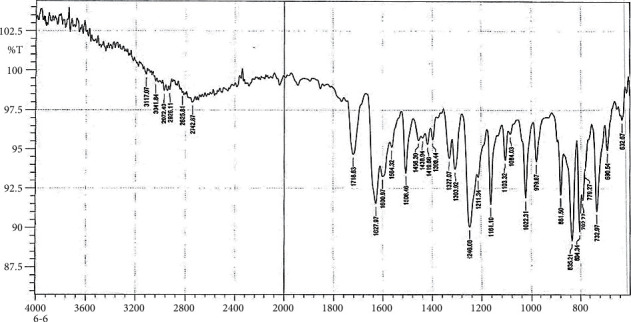
Fourier Transform Infrared Spectrum of C6.

The FTIR peak characters of C5 and C6 compounds appear in Figures 4 and 5. The *υ* (NH) vibrations appear like weak bands at 3068 cm^−1^ and 3117 cm ^−1^, respectively [37].

### 3.5. Proton Nuclear Magnetic Resonance (1H-NMR) Spectra

**Figure 6:**
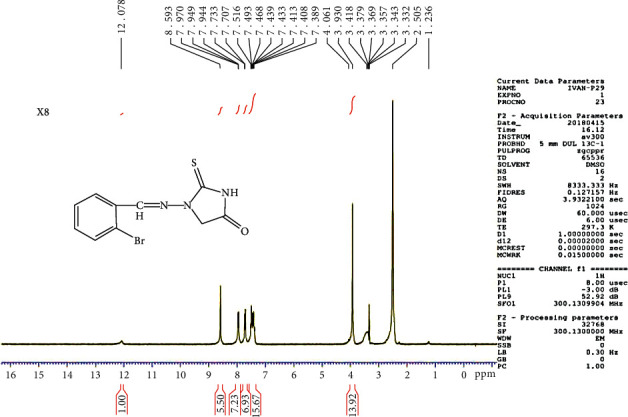
1H-NMR spectrum of the C5 compound.

**Figure 7:**
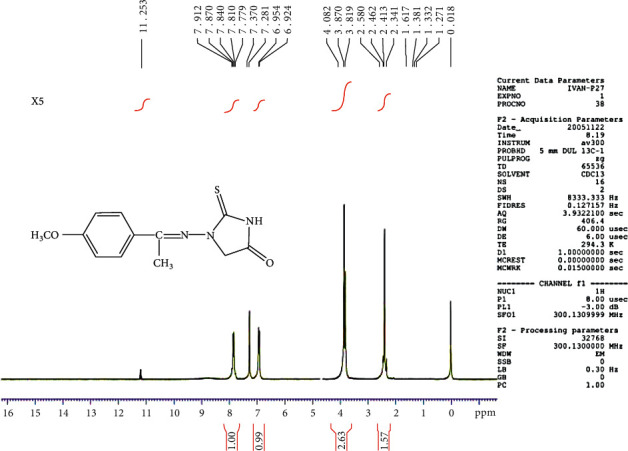
1H-NMR spectrum of the C6 compound.

The 1H-NMR spectrum of the C5 compound shows *δ* (ppm), singlet at 4.06 due to CH2 of imidazolidine, signal at 7.38–7.97 (multiplate for aromatic ring), singlet at 8.59 due to CH=N group, and at 12.07 (singlet for NH) (see Figure 6). The 1H-NMR spectrum of the C6 compound shows *δ* (ppm), singlet at 2.34 due to the methyl group, singlet at 4.08 due to CH2 of imidazolidine, signal at 6.92–7.91 (multiplate for aromatic ring), singlet at group, and at 11.25 (singlet for NH) (see Figure 7).

### 3.6. Bacterial Isolation and Biofilm Formation

**Figure 8:**
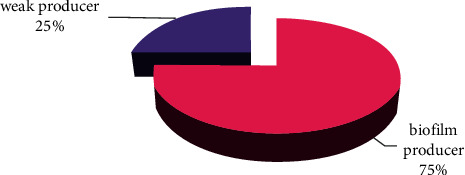
Biofilm production: qualitative assessment by adherence of *S. aureus* clinical isolates to microtiter plates; the degree of the biofilm was interpreted as weak, moderate, and strong biofilm formation as qualified in [28].

The percentage of *S.aureus* isolates from nosocomial-infected patients was 20%; all of these isolates were observed to form biofilms by the M.T.P technique. Strong biofilms were formed by 6 and moderate biofilms by 3 (both strong and moderate accounted for 75%). Weak biofilms were seen in 3 (25%) isolates [28] (see Figure 8, Tables 3 and 4). The biofilm of *S*. *aureus* is one of the major virulence factors that contributes to the environmental success of this pathogen in hospitals [39]. In addition, the formation of biofilms has an association with antibiotic resistance in both phenotypic and genotypic assays of clinical *S. aureus* isolates [40–43].

### 3.7. Antibiotic Susceptibility

All isolated *S. aureus* (12) from this study were multidrug-resistant with different antibiotic-resistant patterns. Multiple antibiotic resistance patterns were observed for Ceftazidime (CAZ30 *µ*g), Piperacillin (PRL30 *µ*g), Azithromycin, (AZM30 *µ*g), and Amoxicillin/Clavulanic acid (AMC 20/10 *µ*g), while 75% were sensitive to Imipenem (IPM 30 *µ*g) (see Tables 4 and 5). The sensitivity was variable for the remaining 6 antibiotics: Ciprofloxacin (CIP 10 *µ*g), Levofloxacin (Lev5 *µ*g), Amikacin (AK 30 *µ*g), Topromycin (TOB5 *µ*g), and Vancomycin (VA 30 *µ*g). The results were determined by describing the resistance of the bacteria (*R*) and the sensitivity of the bacteria (*S*). The inhibition zone diameter was measured and compared to that stated in CLSI.

*S. aureus* vancomycin resistance was 58.33%, and variable levels of vancomycin resistance were indicated. The variability among countries is attributed to the nature of the healthcare system, the method of use, and the number of years used [44]. Antibiotic resistance of *S. aureus* was mainly observed in biofilm producer strains [45, 46]. In connection to that, the key characteristic of continuing any infection is that when the bacterial biological membrane possesses protection from the host immunity and resistance to the antibiotic treatments, therefore, for these antibiotics to be effective, it is necessary to be used in higher concentrations (500–2000 times) [47]. Moreover, S*. aureus* clinical isolates can easily acquire the antibiotic resistance gene from the environment [48, 49]. Thus, emphatically, the World Health Organization (WHO) has declared a list of antibiotic resistance bacteria in a way which requires new antibiotics urgently. Most antibiotic resistance pathogens include hospital-acquired bacteria including methicillin-resistant (MRSA), vancomycin-intermediate, and resistant *Saphylococcus aureus* (VISA) (VRSA), which can cause chronic infections; in addition to that, *S. aureus* can spread easily in the environment. Therefore, these bacteria bring attention to find new antibacterial therapies [50, 51]. According to [52], 2% of people are asymptomatic carriers of MRSA, which can infect the delimited immunity of individuals and become problematic. Furthermore, the VISA and VRSA are highly resistant to antibiotics and become a challenge to treat. However, in severe infections, the VISA-resistant mechanism is ambiguous [53].

### 3.8. Immunological Markers

#### 3.8.1. Serum Level of Immunoglobulin (Ig) and Complement in the Sera of Patients (Infected by *S. aureus*)

The level of IgM and IgA appeared normal in the sera of patients infected with strains (produced and non-produced biofilms) (*P* > 0.001), while the IgG level was significantly elevated in patients infected with strains (produced and non-produced biofilms) (see Table 6). Also, the components of the complement (C3) level appeared normal in patients infected with isolates-produced biofilm compared to non-produced biofilm isolates, respectively (*P* > 0.001), while the levels of complement components (C4) were elevated for both groups (formed and non-formed biofilms) (see Table 6). Generally, IgG and IgA levels appeared higher in patients' sera infected with strains that produced biofilms compared with those who were infected with strains that did not produce biofilms; thus, it predicates the outcome that bacteria-produced biofilms cause chronic infections as the serum IgG or IgA elevated and the complement-dependent human neutrophil phagocytosis was not affected [54–56] *S.aureus* biofilm produces staphylococcal complement inhibitor (SCIN) that binds and stabilizes C3 convertase [57, 58]. Besides all that, complements may be activated via immunoglobulins binding to the extracellular surface of the biofilms or surface molecules, prompting complement enactment and enhanced bacterial opsonization in a classical pathway that begins strongly with IgM immunoglobulins binding to the bacterial surface molecules following complement activation to form C3 convertase that cleaves the C3 protein [59–61]. *S. aureus* biofilms escape the immune detection by evading Toll-like receptors (TLRs); TLR4 acts as a ligand for bacterial lipopolysaccharide, and TLR9 for oligonucleotides of cytosine and guanine with phosphodiester backbone (CpG); oligonucleotide and bacterial DNA sugar backbone cause complement activation [62]. The TLR4 and TLR9 activation strongly triggers plasma interleukin-6 (IL-6), tumor necrosis factor-alpha (TNF- alpha), and IL-1beta, thus insinuating that the inflammatory response of IL-1*β* and TLR families is critical to initiate an immune response; IL-1*β* play a role in T-cell-dependent antibody production and increases the bacterial biofilm formation [63–65]. In general, the levels of IgM and C3 were normal in both the groups, but the IgM values were higher in patients' sera infected with strains that did not produce biofilms compared with those infected with strains that produced biofilms, as IgM may switch to IgG antibody by T-independent antigens in chronic infections [66]. The immunoglobulins might be suppressed from immune recognition by the biofilm's extracellular matrix or host immune cell [67]. *S.aureus* blocks IgA and inhibits all three pathways of complement activation by producing a superantigen like-protein (SSL7), which binds with the C5 protein complement in the serum [68, 69], The C4 complement levels increased in all types of *S. aureus* biofilm formations, which can be assumed is due to the immune response against *S. aureus* biofilm infection triggering lectin pathway-dependent C4 turnover, which is initiated by lipoteichoic acid complexes with serum L-ficolin [59, 70], and by binding serum “recognition” lectin, Mannan Binding Lectin (MBL) with human serum glycans, IgG- G0 [71, 72]. Subsequently, the levels of these immunoglobulins increased [73]. The IgG enhances all complement attack via cleaving C3 and C4, take-up onto tissues and target cells, accordingly raised the level of IgG in sera of patients that might be the origin of complement level that propose immunoglobulins can assume function in active therapy in diseases joined via actuation of the classical complement pathway. Additionally, these levels mirrored the innate immunity status of the individual, particularly C3 which is the main segment of the complement pathway, while the degree of C4 was raised; a decrease in C3 level is a decent sign for the alternative pathway complement enactment because of nearby non-obtrusive bacterial infection [74]. Although few studies indicated the immune responses towards bacterial biofilms, the development of the biofilm has a significant part in the avoidance of the host immune defenses; biofilms shield bacteria from antimicrobial peptides; neutrophil phagocytosis; and the deposition of complements and antibodies [4, 75]. This way the biofilm of *S. aureus* has become extremely problematic in the treatment of infections caused by the strains as they can evade the immune system.

### 3.9. Hemagglutinin Assay

*Hemagglutinin* plays a chief part in the devotion of this organism adhesion to a polymer comprising biomaterials [77].  Table 7 illustrates the hemagglutination assay for the 12 isolates and the relationship between hemagglutination-positive reaction (macroscopically and microscopically) and biofilm formation. The positive reaction was observed macroscopically in 6 isolates, while 8 isolates caused the positive reaction microscopically. From these 12 isolates, 6 of them formed strong biofilms, and the positive reaction macroscopically was observed in 4 isolates and microscopically in 5. Among the moderate biofilm formation, which was 3, the positive reaction was observed in 2 isolates macroscopically and 3 isolates microscopically. Those isolates that produced weak biofilms did not agglutinate any of the tested types of erythrocytes. These results indicate the relationship between the formation of biofilms and hemagglutination; biofilm producer strains were able to agglutinate erythrocytes [43]. The mechanism of hemagglutination is due to the attraction of the bacterial polysaccharide charge with the negative charge of the erythrocyte surface [44]. Hemagglutination may play a role in the pathogenesis of infections [10, 44] or may serve as an alternative marker for adherence isolates by microtiter plate assay. The most complicated to treat from biofilm formation adherence are biomaterial-associated infections [77], the extracellular polysaccharide production in Staphylococcus spp. biofilm, polysaccharide intercellular adhesin (PIA), and poly-N- acetylglucosamine (PNAG) that can agglutinate erythrocytes [1, 78]. Thus, Staphylococci that can produce biofilms causes hemagglutination by extracellular polysaccharide; strong biofilm strains that produced the secreted PIA showed hemagglutination [80], and consequently staphylococcus becomes more difficult to treat.

### 3.10. *InVitro* Biocompatibility Evaluation

To assess the clinical potential of the compounds, their effects and antibacterial activity against virulent *S. aureus* clinical isolates have been evaluated. The compounds' effective dose that reduced the bacterial growth was from 31.25 *μ*g/ml to 500 3 *μ*g/mL.

### 3.11. Minimal Inhibitory Concentrations (MICs) of the New Hydantoin Derivatives

The MIC value of the new hydantoin derivatives (C5 and C6) *in vitro* against the 12 virulent *S. aureus* isolates are reported in Tables 8 and 9:

- C5 and C6 compounds showed excellent activity against virulent *S. aureus* at a concentration of 125 *μ*g/mL; C5 inhibited 7 isolates and C6 inhibited 8 isolates.
- The MIC value for compound C5 was 31.25 *μ*g/mL in 2 isolates, and the MIC was 62.5 *μ*g/mL in 3 isolates.
- The MIC value for C6 compound was 31.25 *μ*g/mL in one isolate, and 62.5 *μ*g/mL in 2 isolates. The MIC was 250 *μ*g/mL in 2 isolates
- For both the compounds, the MICs were 500 *μ*g/mL and the MIC 250 *μ*g/mL for C5 was not assessed.
- Finally, the new hydantoin compounds were not concentration dependent.

Generally, the compounds showed potentiating properties at concentrations from 31 to 125 µg/mL. Thus, these compounds have more potential than previously reported anti-S.aureus activity MIC values [80–82]; in addition to the tested bacteria, there are other virulent strains. Hydantoin derivatives are small molecules that have moderate antimicrobial activity which may be essential for reducing antibiotic resistance in the long run, besides being potent and having rapid antimicrobial activity against pathogenic bacterial strains [83]. The mechanisms on the bacterial membrane are similar to natural host defense peptides and these hydantoin compounds also reduce the problem of MRSA [84], VISA, and VRSA bacterial resistance under the tested conditions.

### 3.12. Time-Kill Assay

**Figure 9:**
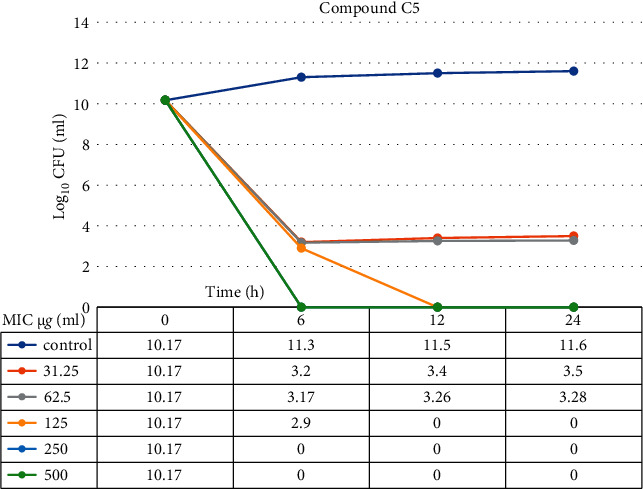
Time-kill kinetic of the C5 compound from 1X to 16X of MIC for 6, 12, and 24 hours against *S. aureus* isolates.

**Figure 10:**
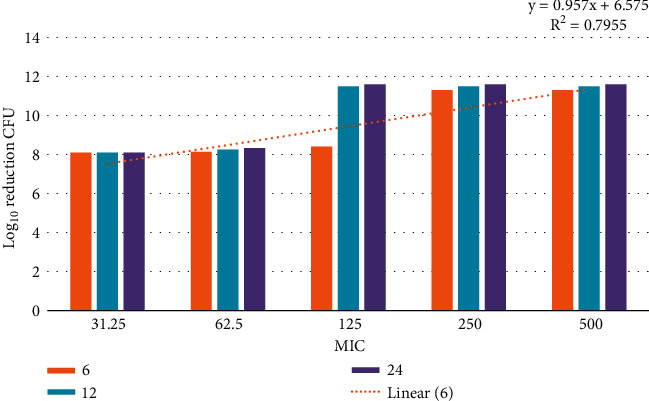
IC50 for the C5 compound from 1X to 16X of MIC for 6, 12, and 24 hours against *S. aureus* isolates.

**Figure 11:**
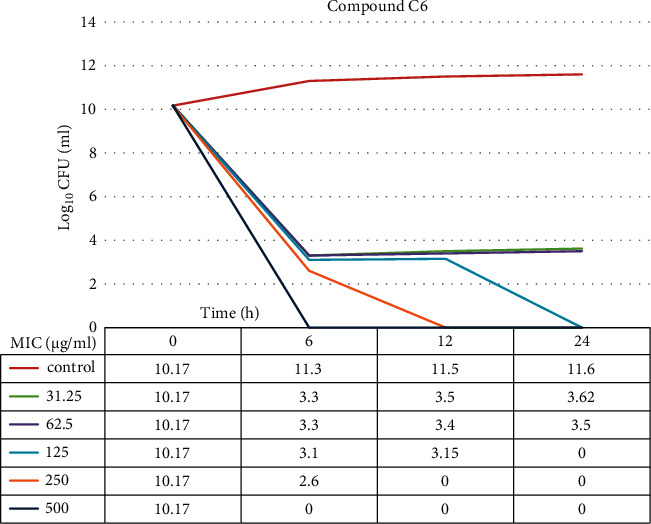
Time-kill assay of the C6 compound from 1X to 16X of MIC for 6, 12, and 24 hours against *S. aureus* isolates.

**Figure 12:**
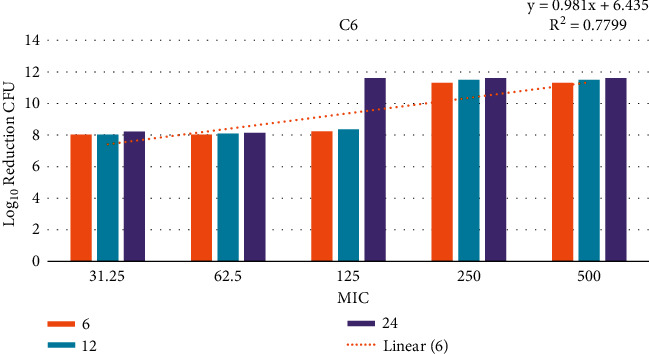
IC50 of the C6 compound from 1X to 16X of MIC for 6, 12, and 24 hours against *S. aureus* isolates.

The data demonstrated in Figures 9–12 refer to the effect of the different concentrations of C5 and C6 compounds (31.25 *µ*g/ml (1X) to 500 *µ*g/ml (16X)) on the growth of bacteria. All treated cultures were reduced in the CFU count of bacteria in comparison with the initial inoculum. The time-kill kinetics of the new hydantoins against the test organisms (*S. aureus* at test concentrations) showed a reduction in the number of viable cells over the 6, 12, and 24 hours compared to the control. Furthermore, the resultant effect of the compounds from incubating the bacteria at 1, 2X MICs was a rapid reduction in the average of the 7log10 reduction (99.99% inhibition). The reduction in the cell counts between 12 and 24 h of incubation period showed that the compounds were highly bactericidal. Regarding the C5 compound, the bacterial colonies were totally wiped out after incubating for 6 h at concentration of 8, 16X MICs and for 12h at 4X MICs, and the IC50 of the compound was 46.7892 ± 2.51e + 04 (5.365e + 04%). With the C6 compound, bacterial colonies were totally wiped out after incubation for 6, 12, and 24 h at the concentration of 16X, 8X, and4 MICs, respectively, and the IC50 of the compound was 55.0085 ± 4.758e + 04 (8.649e + 04%). The results of the time-kill profiles for the tested bacteria and antibacterial assays determined that time-kill depends on the compounds' molecular docking.

### 3.13. Biofilm Inhibition Activity

The antibiofilm activity of the compounds, C5 and C6, were determined toward six strains, selected in the present study due to their strong biofilm-forming ability.

#### 3.13.1. Effect of Compound C5[3-((2- Bromobenzylidene) amino)-Thioxoimidazolidin-4-One]

The highest level of inhibition was 99.19% at 62.5 *μ*g/mL in one isolate, followed by 91.63% and 90.74% at 31.25 *μ*g/mL, while two isolates had an inhibition close to 89% at 62.5 *μ*g/ml (see Table 10). The lowest inhibitory effect was 82.38% and appeared in one isolate. We found the effect value of subMICs was different in compound C5 against *S. aureus* isolates; the MICs' difference ranged from 31.25 *µ*g/mL to 62.5 *µ*g/mL. Consequently, the new hydantoin compound was not concentration dependent and will need further investigation for the mechanism of action. Moreover, the results showed significant differences (*P* < 0.05) between the inhibition rate of biofilms and untreated biofilms. The biofilm formation decreased significantly as compared with untreated biofilms of isolates 9 and 8 at concentration 62.5 *μ*g/mL, and isolate 11 at 31.25 *μ*g/mL. There were no significant differences in the biofilm inhibition of C5 concentration of isolates 2, 6 at 62.5 *μ*g/mL, and isolate 3 at 31.25 *μ*g/mL.

#### 3.13.2. Effect of Compound C6 [3-((4- Methoxybenzylidene) Amino)- 2-Thioxoimidazolidin-4- One]

Compound C6 showed significantly high percentages of inhibition (93.95%) at 125 *μ*g/mL and 62.5 *μ*g/mL (see Table 11); however, isolate 11 exhibited low activity (75.35%). Biofilms' formation was significantly reduced as compared to the untreated biofilms of isolate 9 at the tested concentrations 125 *μ*g/mL, and isolates 2 and 8 at 62.5 *μ*g/mL of C6 compound. There were no significant differences in reduction in isolates 3 and 11, at concentrations 62.5 *μ*g/mL and isolate 6 at 125 *μ*g/mL.

This was validated by ANOVA with a significance level of 95% and mean comparison with an *α* error of 0.05. Also, the results showed that the effect value of subMICs was different in compound C6 against *S. aureus* isolates, which ranged from 62.5 *µ*g/mL to 125 *µ*g/mL. Hence, the new hydantoin compound was not concentration dependent. Moreover, the results illustrated a significant difference (*P* < 0.05s) between the inhibition rate of treated biofilms and untreated biofilms. Consequently, the potential of exhibiting antibiofilm activity against biofilms of *S. aureus* is more by these compounds compared to the previous reports (it inhibited 40% *S. epidermidis* ATCC 12228 biofilm) [82]. So far, only a few studies have been found about inhibited biofilms by hydantoin compounds.

### 3.14. Effect of the New Hydantoin Compounds on *S*. *aureus* Hemagglutination

In this study, the C5 and C6 hydantoin compounds evidenced hemagglutination inhibition against five of the six virulent bacteria (see Tables 12 and 13). The compound C5 manifested antihemagglutination effects in the MIC range of 31.25–62.5 *µ*g/mL concentrations, while C6 showed effects in the range of 62.5–125 *µ*g/mL concentrations. However, the compounds' concentration of the hemagglutination reduction was not uniform, which was determined using six biofilm strong formation strains that were selected in the present study based on their subMIC biofilm inhibition ability. *S. aureus* strain number 6 has no antihemagglutination effect at a concentration 125 *µ*g/mL in the presence of compound C6, which may be due to the inhibition effect that depends on appropriate compound structure with bacterial strain type. Erythrocytes' hemagglutination is an essential character of *S. aureus* pathogenesis, which is associated with biofilm creation adherence and it is critical for human bacteremia [33]. Most strains of *S. aureus* express binding proteins, fibronectin and fibrinogen, and the clumping factor responsible for bacterial attachment, blood clumping, intravascular hemolysis, and damaged tissue [85]. The data obtained (see Tables 11 and 13) clearly show a relationship between the interaction of the hydantoin compounds with the erythrocyte membrane and their antihemagglutination activity; in connection with these data, the new hydantoin compounds can inhibit the first step in infection establishment. There is a strong association between biofilm formation and hemagglutination-positive strains through adherence factors [86], and as the new hydantoin compounds C5 and C6 have been found to show a strong effect on those factors, this evidence approves the use of the new compounds to prevent biofilms established and removed from medical devices. The most important step to fight bacterial pathogens is to discover some compounds that are active against persister cells that are tolerant to antibiotics [87, 88]; MRSA, VISA, and VRSA are known to form persister cells that lead to inflammation and chronic diseases, indicating that the hydantoin compounds exhibited efficacy in eradicating these strains, which is an approach to improve the effectiveness of drugs.

Moreover, MRSA uses 30 efflux pumps that encode chromosomally and on plasmids to resist antibiotics. Particularly, the NorA efflux pump in *S.aureus* was related to virulence, biofilm formation, and resistance to diverse drugs, especially fluoroquinolone (e.g., ciprofloxacin), quinolones, verapamil, and omeprazole, in addition to biocides, dyes, quaternary ammonium compounds, and antiseptics [89–92]. Therefore, the NorA pump and other efflux pumps are attractive targets to many studies [93–98]. The present study along with previous studies [93–98] showed that the potential antibacterial antibiofilm activities as well as the inhibited NorA efflux pump were because of the compounds' molecular docking mechanisms. The compounds' activity correlated with physicochemical properties, such as positive charge, aromatic ring, and hydrophobic interactions of the compounds with the efflux pump, which is mediated by hydrogen bonds. This interaction controls the compounds' stabilization and association with decreasing bacterial resistance. As a result, functional ligands-based pharmacophore modeling is the principle for the discovery of potential drugs against bacterial resistance [96]. Other studies suggest that lipophilic compounds' character is fundamental to bacterial resistance reduction, which leads to cell membrane alteration, damage, or pore formation. The lipophilic properties depend on hydrophobicity and hydrogen binding rotation [99, 100]. The hydrogen bond formed between the ligand and the receptor is energy dependent and plays a main role in the incorporation. The tested compounds (C5 and C6) reduced *S. aureus* MICs, and were referred to interreact with the resistance mechanisms, which are mediated mostly by efflux pumps [94]. Additionally, [101] the study demonstrated that hydantoin compounds played a crucial role in inhibiting the AcrAB pump activity, and that they affect bacterial membranes, similar to host defense peptides, which are expressed naturally by body responses. Hydantoin compounds rapidly prevented the growth of bacterial pathogens and MRSA; besides, they have in vivo efficacy more than vancomycin by eliminating bacteria, repressing inflammation, and opening new ways for generating potential compounds that are able to inhibit pump activity [1].

## 4. Conclusions

The results demonstrate that all the tested compounds (C5 and C6) prevented biofilm formation, but their effectiveness varied. Overall, all the compounds were able to strongly prevent hemagglutination; besides, the effectiveness of the compounds has not depended on concentration. [101] noted that the activity of hydantoin compounds depends on the size and the group of substitution. Hence, the previous studies [1, 2, 102] along with the present study indicate that the hydantoin compounds' activity depends on the compatibility of the chemical structure of the compounds with the bacterial type. Nonetheless, modification of the hydantoin compounds could improve the activity which will be crucial in exploring different and more efficient drugs. Besides all that, we have found that clinical *Staphylococcus aureus* biofilm-producing strains produce extracellular polysaccharides that play an important role in the recognition by immunoglobulins to activate complement factors and elicit strong hemagglutination that consequently becomes more difficult to be treated with antibiotics.

## Figures and Tables

**Table 1:** Physical properties and yield percentage of compounds S5, S6, C5, and C6.

Compound symbol	Molecular formula	Compound name	M.P°C	Color	Yield %
S5	C_8_H_8_BrN_3_S	2-(2-Bromobenzylidene) hydrazine-1- carbothioamide	190–192	White	85
S6	C_9_H_11_N_3_OS	2-(4-Methoxybenzylidene) hydrazine-1- carbothioamide	165–168	Pale brown	90
C5	C_10_H_8_BrN_3_OS	3-((2-Bromobenzylidene)amino)-2-thioxoimidazolidin-4- one	246–248	White	87
C6	C_11_H_11_N_3_O_2_S	3-((4-Methoxybenzylidene) amino)- 2- thioxoimidazolidin-4- one	273–275	Pale brown	93

**Table 2:** Classification of bacteria adherence by the M.T.P method [28].

Optical density	Adherence	Biofilm formation
<0.120	Non	None/weak
0.120–0.240	Moderate	Moderate
>0.240	Strong	High

**Table 3:** In vitro biofilm production by *S. aureus* clinical isolates by the M.T.P method; the degree of the biofilm was interpreted as weak, moderate, and strong biofilm formation as qualified according to optical density (OD) [27].

No. of isolates	OD rate of the test sample	OD rate of the test sample - OD of control
Control (negative)	0.119	
*S. aureus 1*	0.223	0.104
*S. aureus 2*	0.449	0.330
*S. aureus 3*	0.362	0.243
*S. aureus 4*	0.211	0.092
*S. aureus 5*	0.320	0.201
*S. aureus 6*	0.410	0.291
*S. aureus 7*	0.174	0.055
*S. aureus 8*	0.560	0.441
*S. aureus 9*	0.402	0.283
*S. aureus 10*	0.315	0.196
*S. aureus 11*	0.487	0.368
*S. aureus 12*	0.291	0.172

**Table 4:** The percentage of antibiotic resistance of virulent *S. aureus* isolates was detected by the diffusion method.

Antibiotic	Percentage of resistance	Kind of antibiotic	Percentage of resistance
Ceftazidime	100	Vancomycin	58.33
Piperacillin	100	Amikacin	50
Azithromycin	100	Levofloxacin	41.67
Amoxicillin/Clavulanic acid	100	Ciprofloxacin	41.67
Topromycin	66.68	Imipenem	25

**Table 5:** Sensitivity of the 12 *S. aureus* isolates to antibiotics.

No. of isolates	Antibiotics^*∗*^										No. of antibiotic resistance group
	CAZ	PRL	AZM	AMC	TOB	VA	AK	Lev	CIP	IPM	
*S. aureus 1*	R	R	R	R	S	R	S	R	R	S	7
*S. aureus 2*	R	R	R	R	R	S	R	S	S	R	7
*S. aureus 3*	R	R	R	R	R	R	S	S	S	S	6
*S. aureus 4*	R	R	R	R	S	S	R	R	R	S	7
*S. aureus 5*	R	R	R	R	R	R	S	R	R	S	8
*S. aureus 6*	R	R	R	R	R	R	S	S	R	R	8
*S. aureus 7*	R	R	R	R	R	S	R	S	S	S	7
*S. aureus 8*	R	R	R	R	R	R	R	S	S	R	8
*S. aureus 9*	R	R	R	R	R	S	S	R	R	S	7
*S. aureus 10*	R	R	R	R	S	R	R	S	S	S	6
*S. aureus 11*	R	R	R	R	S	S	R	S	S	S	5
*S. aureus 12*	R	R	R	R	R	R	S	R	S	S	7

*S* = sensitive to antibiotic *R* = resistant to antibiotic. ^*∗*^CAZ: ceftazidime (30 *µ*g), PRL: piperacillin (30 *µ*g), AZM: azithromycin (30 *µ*g), AMC: amoxicillin/clavulanic acid (20/10 *µ*g), IPM: imipenem (30 *µ*g), CIP: ciprofloxacin (10 *µ*g), Lev: levofloxacin (5 *µ*g), AK: amikacin (30 *µ*g), TOB: topromycin (5 *µ*g), and VA: vancomycin (30 *µ*g).

**Table 6:** Complement levels and immunoglobulin mean values in patients' sera with *S. aureus* (formed and non-formed biofilms) infection.

Immunoglobulin (mg/dl)	Patients sera infected by *S. aureus* (*M* ± *SD*)		Normal value	*P*-value
	Produced biofilm (No. = 9)	Non-produced biofilm (No. = 3)		
IgG (mg/dl)	1424 ± 722.320	1412.3 ± 485.320	710–1520	(*P* < 0.001) Significant
IgM (mg/dl)	80.13 ± 34.20	113.5 ± 31.30	40–250	(*P* > 0.001) not significant
IgA (mg/dl)	219.8 ± 9.185	190.12 ± 81.013	90–310	(*P* > 0.001) not significant
Complements				
C3 (mg/dl)	110 ± 25.6	110 ± 35.44	84–193	(*P* > 0.001) not significant
C4 (mg/dl)	51.7 ± 15.07	52 ± 13.23	20–40	(*P* > 0.001) not significant

IgG: Immunoglobulin G, IgM: Immunoglobulin M, IgA: Immunoglobulin A, C3: complement 3, C4: complement 4, *M* = mean, and SD = standard deviation.

**Table 7:** The relationship between biofilm formation and hemagglutination assay in *S. aureus*.

Biofilm formation	No. of isolates	Hemaglutination assay			
		Macroscopically		Microscopically	
		+	−	+	−
Strong +	6	4	2	5	1
Moderate +	3	2	1	3	0
Weak-	3	—	3	—	3
Total No.	12	6	6	8	4

Note: (+): growth, Number (MIC), (−): no growth.

**Table 8:** MIC values of the new hydantoin compounds, C5 and C6, against the 12 virulent *S. aureus* isolates.

No. of isolates	MIC (*µ*g/mL)	
	Compound (C5)	Compound (C6)
*S. aureus 1*	31.25	62.5
*S. aureus 2*	125	125
*S. aureus 3*	62.5	125
*S. aureus 4*	62.6	125
*S. aureus 5*	125	125
*S. aureus 6*	125	250
*S. aureus 7*	31.25	62.5
*S. aureus 8*	125	125
*S. aureus 9*	125	250
*S. aureus 10*	125	125
*S. aureus 11*	62.5	125
*S. aureus 12*	125	125

**Table 9:** MIC values of the new hydantoin compounds, C5 and C6, against the 12 virulent *S. aureus* isolates.

Compounds code	MIC (*µ*g/mL)				
	31.25	62.5	125	250	500
C5	2 isolates	3 isolates	7 isolates	0	0
C6	12 isolates	2 isolates	8 isolates	2 isolates	0

**Table 10:** The biofilm formation and biofilm inhibition activity of the hydantoin compound C5 in *S. aureus* isolates according to subMIC.

Isolates	SubMIC	Biofilm formation	Inhibition of biofilm	% of inhibition
9	62.5	0.283	0.009°	99.19
3	31.25	0.243	0.094	91.63
11	31.25	0.368	0.104°	90.74
8	62.5	0.441	0.120°	89.32
6	62.5	0.291	0.121	89.23
2	62.5	0.330	0.198	82.38
LSD value	—	—	0.218	

^*∗*^(*P* < 0.05). ° Significant biofilm decreased compared with untreated biofilm. *t* = 5.7408; df = 1. Standard error of difference = 0.038. 95% confidence interval of this difference: from 0.13359 to 0.30307; confidence interval: 0.21833.

**Table 11:** The biofilm formation and biofilm inhibition activity of the hydantoin compound C6 in S. *aureus* isolates according to SubMIC.

No. of isolates	SubMIC	Biofilm formation	Inhibition of biofilm	% of inhibition
9	125	0.283	0.068°	93.95
2	62.5	0.330	0.126°	88.79
3	62.5	0.243	0.129	88.52
8	62.5	0.441	0.149°	86.74
6	125	0.291	0.186	82.45
11	62.5	0.368	0.277	75.35
LSD value	—	—	0.168	

^*∗*^(*P* < 0.05). ° Significant biofilm inhibition compared with untreated biofilm. *t* = 5.2581; df = 5. Standard error of difference = 0.032. 95% confidence interval of this difference: From 0.08544 to 0.24889; confidence interval: 0.16717.

**Table 12:** Effect of the hydantoin compound C5 on hemagglutination activity in relationship with S. aureus biofilm formation.

No. of isolates	SubMIC	Hemaglutination assay			
		Microscopically		Macroscopically	
		Without C5	With C5	Without C5	With C5
2	62.5	+	−	+	−
3	31.25	+	−	+	−
6	62.5	+	−	+	+
8	62.5	+	−	+	−
9	62.5	+	−	+	−
11	31.25	+	−	+	−

**Table 13:** The effect of the hydantoin compound C6 on the hemagglutination activity in relationship with *S. aureus* biofilm formation.

No. of isolates	SubMIC	Hemaglutination assay			
		Microscopically		Macroscopically	
		Without C6	With C6	Without C6	With C6
2	62.5	+	−	+	−
3	62.5	+	−	+	−
6	125	+	+	+	+
8	62.5	+	−	+	−
9	125	+	−	+	−
11	62.5	+	−	+	−

## Data Availability

All data used to support the findings of this study are included within the article.

## Abbreviations

AK: Amikacin
AMC: Amoxicillin/clavulanic acid
ANOVA: Analysis of variance
API: Analytical profile index
ATCC: American-type culture collection
AZM: Azithromycin
BHI: Brain heart infusion broth
BSA: Bovine serum albumin
C3: Complement component 3
C4: Complement component 4
C5: 3-((2-Bromobenzylidene)amino)-2- thioxoimidazolidin-4-one C6: 3-((4-methoxybenzylidene)amino)- 2-thioxoimidazolidin-4-one
CAZ: Ceftazidime
CIP: Ciprofloxacin
CLSI: Clinical and laboratory standards institute
CpG: Oligonucleotides of cytosine and guanine with phosphodiester backbone
FTIR: Fourier Transform Infrared Spectroscopy
IgA: Immunoglobulin A
IgG: Immunoglobulin G
IgM: Immunoglobulin
MIL: Interleukin
IPM: Imipenem
Lev: Levofloxacin
M.T.P: Micro titration plates approach
MBL: Mannan binding lectin
MIC: Minimal Inhibitory concentration
MRSA: Methicillin-resistant *Staphylococcus aureus*
OD: Optical density
PBS: Phosphate-buffered saline
PIA: Polysaccharide intercellular adhesin
PNAG: Poly-*N*-acetylglucosamine
PRL: Piperacillin
S5: 2-(2-Bromobenzylidene) hydrazine-1-carbothioamide
S6: 2-(4-METHOXYBENZYLIDENE) hydrazine-1-carbothioamide
SCIN: Staphylococcal complement inhibitor
SRID: Single radial immunodiffusion SSL7: superantigen like-protein
TLR: Toll-like receptor
TNF-alpha: Tumor necrosis factor-alpha
TOB: Topromycin
TSB: Tryptic soy broth
VA: Vancomycin
VISA: Vancomycin-intermediate *Staphylococcus aureus*
VRSA: Vancomycin-resistant *Staphylococcus aureus*
WHO: World Health Organization.
